# Missed Gastroesophageal Injuries During Antireflux Surgery: Infrequent but Catastrophic Complications

**DOI:** 10.3390/jcm14134577

**Published:** 2025-06-27

**Authors:** Arianna Vittori, Andrés R. Latorre-Rodríguez, Andrew Keogan, Jasmine Huang, Lara Schaheen, Ross M. Bremner, Sumeet K. Mittal

**Affiliations:** 1 Norton Thoracic Institute, St. Joseph’s Hospital and Medical Center, Phoenix, AZ 85013, USA; arianna.vittori@commonspirit.org (A.V.); andres.latorre@commonspirit.org (A.R.L.-R.); andrew.keogan@commonspirit.org (A.K.); jasmine.huang@commonspirit.org (J.H.); lara.schaheen@commonspirit.org (L.S.); ross.bremner@commonspirit.org (R.M.B.); 2 Department of Surgery, Oncology and Gastroenterology, School of Medicine, University of Padua, 35128 Padova, Italy; 3 Grupo de Investigación Clínica, Escuela de Medicina y Ciencias de la Salud, Universidad del Rosario, Bogotá D.C., Colombia; 4 School of Medicine, Creighton University, Phoenix, AZ 85012, USA

**Keywords:** hiatal hernia, antireflux, fundoplication, perforations, leaks, surgical complications

## Abstract

**Background**: Laparoscopic antireflux surgery (LARS) is widely used to treat gastroesophageal reflux disease (GERD). Iatrogenic gastroesophageal injuries, when recognized intraoperatively, can be managed without major consequences, whereas undetected injuries presenting as postoperative leaks are associated with high morbidity and mortality. Despite their complexity, research on post-LARS leaks is scant. We aim to describe the diagnosis and management of such injuries at a tertiary referral center. **Methods**: We describe a single-center case series of patients referred for gastroesophageal perforations after LARS. Patients were identified through the personal records of surgeons at our institution. A narrative literature review was conducted to summarize publications on the topic. **Results**: Five patients (four female [80%]; median age, 73 years [IQR, 67–74]) were included. The median time between LARS and clinical presentation was 2 (IQR, 1–8) days (range 1–15 days). The most frequent symptoms were shortness of breath (all five patients) and pain (three [60%] patients). All patients presented with hypoxia, and four (80%) patients presented with sepsis. Two (40%) patients underwent primary repair, and three (60%) required limited esophagogastrectomy without immediate reconstruction. All patients required both thoracic and abdominal exploration, and all of them experienced significant postoperative complications (Clavien–Dindo ≥ 3). The median hospital stay was 58 days (IQR, 34–59). At a median follow-up of 14 months (IQR, 6–28), all patients were alive. **Conclusions**: Although infrequent, gastroesophageal perforation after LARS often requires complex surgical interventions and prolonged hospital stays. Additional efforts should focus on prevention and early recognition.

## 1. Introduction

Gastroesophageal reflux disease (GERD) is one of the most common gastrointestinal disorders globally, with an estimated prevalence of approximately 20% in Western countries and a rising global incidence [1]. Laparoscopic antireflux surgery (LARS) is the gold standard surgical treatment for GERD [2,3]. It has gained widespread recognition for its safety and effectiveness, given its low complication rates and excellent long-term outcomes [4,5,6]. However, as with any major surgical procedure, the risk of adverse events remains. The most serious and potentially life-threatening are missed gastroesophageal injuries presenting postoperatively, a rare complication reported in 0.1–0.3% of patients undergoing primary antireflux surgery in the modern era [7,8]. Despite its high morbidity, discussions regarding iatrogenic perforations missed during LARS and diagnosed postoperatively remain limited in the surgical literature, with just a few series published in the 1990s [9,10]. This gap in evidence undermines the development of standardized protocols for both prevention and management of affected patients. The aim of our study is to describe the presentation, treatment, and outcomes of leaks diagnosed postoperatively and managed at a tertiary referral center. Further, we conducted a thorough literature review of all described cases of iatrogenic perforations diagnosed after LARS to synthesize the available evidence.

## 2. Methods

### 2.1. Study Design and Settings

This single-center, retrospective, observational case series analyzed de-identified data from consecutive patients with esophageal or gastric perforations after antireflux surgery who were transferred for acute management to a high-volume referral center for foregut diseases in the southwestern United States. The Institutional Review Board of St. Joseph’s Hospital and Medical Center, Phoenix, AZ approved this study under the Foregut Umbrella Protocol (PHXU-21-500-136-73-18, project approval date: 28 January 2025). Due to the study design, written patient consent was waived. The Preferred Reporting Of CasE Series in Surgery (PROCESS) 2023 guidelines were followed to ensure transparency of the results and overall quality of the manuscript [11]. This study was preregistered on the Open Science Framework (registration: https://doi.org/10.17605/OSF.IO/DT5SY, date: 24 March 2025).

### 2.2. Study Population

This study included all patients who were transferred to our center with gastroesophageal perforation after antireflux surgery. We excluded (i) patients who underwent Collis gastroplasty or Roux-en-Y reconstruction; (ii) perforations recognized and treated during LARS; (iii) patients diagnosed with perforation after intense retching or vomiting or those presenting with acute re-herniation leading to subsequent strangulation and perforation; (iv) patients transferred for suspected perforation but who did not have a confirmed leak; (v) cases of perforation presenting more than 30 days after surgery, as the likelihood of this condition being secondary to iatrogenic causes was considered low; and (vi) patients treated at our institution for late complications, such as persistent pleural/mediastinal abscess after LARS.

### 2.3. Data Source and Variables

Cases were identified from the personal records of the surgeons at our institution and retrospectively analyzed. Patient chart extraction was manually performed from a Cerner database (Oracle, North Kansas City, MO, USA). Information gathered included patient demographics, clinical characteristics, details of index surgery, surgical and medical management of perforations, and outcomes, such as hospital stay, additional complications, and mortality. At the follow-up, nutrition status, digestive function, activity status, pain, and self-perceived well-being [12] were assessed using a Likert-type scale (4, very good; 3, good; 2, neutral; 1, poor; 0, very poor). Only non-identifying information necessary for analysis was included, and any remaining potentially identifying details were excluded from the dataset. All surgical procedures for treating gastroesophageal perforations were performed by experienced surgeons.

### 2.4. Literature Review

An automated search was conducted in MEDLINE (via PubMed) to investigate the management of early (i.e., presenting less than 30 days from surgery) esophageal or gastric perforations secondary to open, laparoscopic, or robotic fundoplications performed for GERD or hiatal hernia. The search included articles from the inception of the database to December 2024, using the following MeSH and free-text keywords: “fundoplication OR antireflux surgery OR hiatal hernia repair” AND “perforation OR leak” AND “management OR treatment.” A total of 390 articles were initially identified. After removing duplicates (n = 81) and non-English publications (n = 19), 290 publications were eligible for further screening. Only articles that provided a comprehensive description of the diagnostic process and treatment strategies for gastroesophageal perforations recognized postoperatively in patients aged 18 years and older were included. Studies involving patients with prior or concomitant bariatric surgeries or undergoing concomitant Collis gastroplasty were excluded. After a second screening by full reading, 7 studies, with a total of 22 cases, were included in this review. The review flowchart is shown in Supplementary Material Figure S1.

### 2.5. Statistical Analysis

Given the descriptive nature of this study, descriptive statistics were employed to summarize the demographic and clinical characteristics of the study cohort and the studies included in the literature review. Categorical variables were expressed as counts and proportions, and continuous data were expressed as medians and interquartile ranges (IQR). Calculations were performed using R version 4.3.2 (R Foundation for Statistical Computing, Vienna, Austria).

## 3. Results

### 3.1. Population Characteristics

Ten patients met the initial criteria; however, five patients were excluded: one patient had a concomitant Collis gastroplasty; one patient was referred after a repair performed outside for persistent mediastinal collection; one patient was transferred for suspected perforation, although the leak was not confirmed; and two patients presented with a postoperative leak despite the perforation being recognized and treated during LARS. Therefore, five patients were included in our study population; none of these cases have been previously described.

Four (80%) patients were female, the median age was 73 years (IQR: 67–74), and the median BMI was 31.60 kg/m^2^ (IQR, 30.30–36.11). The most common comorbidities included hyperlipidemia (n = 4 [80%]) and hypertension and hypothyroidism (n = 3 each [60%]). There were no tobacco smokers or patients with history of substance abuse, though one patient (20%) reported excessive alcohol intake. The study population characteristics are reported in Table 1.

### 3.2. Antireflux Surgery

The indication for surgery in all patients was a large paraoesophageal hernia, which was associated with GERD in two (40%) patients. All procedures were primary elective surgeries. Three surgeries (60%) were laparoscopic and two (40%) were robot-assisted. The fundoplication types included Nissen (n = 3), Toupet (n = 1), and Watson (n = 1). A bougie was used for calibration in three (60%) cases. A mesh was applied in four (80%) patients. Intraoperative endoscopy after completion of the surgical procedure was reported in one (20%) patient. The median operative time was 210 min (IQR, 170–235). Surgical information is summarized in Table 1.

### 3.3. Clinical Presentation and Diagnosis

**Figure 1:**
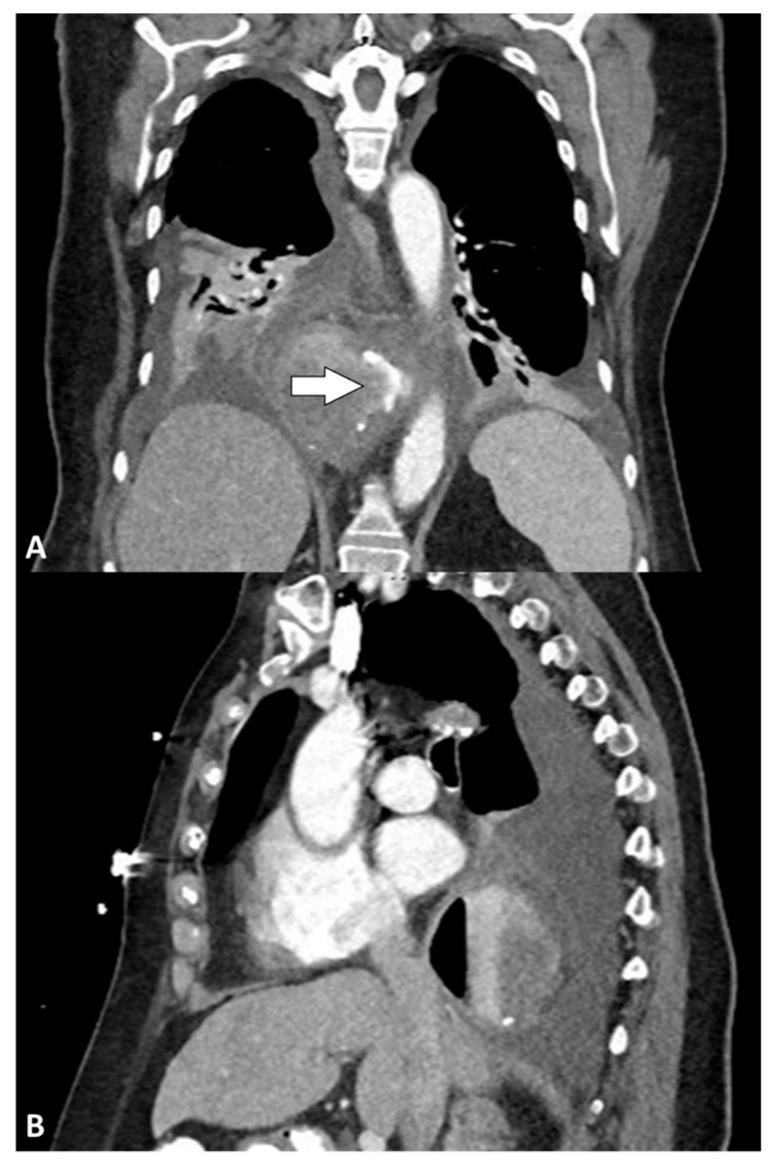
Computed tomography: (**A**) coronal and (**B**) sagittal views showing air and fluid collection surrounding the distal esophagus with oral contrast extravasation (arrow).

The median time between LARS and clinical presentation with leak was 2 (IQR: 1–8) days, ranging from 1 to 15 days. The most frequent symptoms were shortness of breath (all patients) and pain (three [60%] patients). The pain manifested in various forms, including diffuse abdominal pain, thoracic and midscapular pain, and stabbing thoracic pain. All patients presented with hypoxia (i.e., resting SaO_2_ of less than or equal to 95%) [13], and four (80%) required emergent intubation. Septic shock, according to the Sepsis-3 definitions [14], was reported at presentation in four (80%) patients. New-onset atrial fibrillation was observed in one (20%) patient, three (60%) patients had a clinical or radiological diagnosis of subcutaneous emphysema, and three (60%) patients had acute kidney injury at presentation. Notably, fever (i.e., body temperature > 37.5 °C) was absent in all patients. The median white blood cell count was 14.5 × 10^9^/L (IQR, 13.8–16.5), and the rest of the bloodwork was unremarkable. The first diagnostic hypotheses were postoperative leak in two (40%) patients, pneumonia in two (40%) patients, and non-ST-elevation myocardial infarction (NSTEMI) in one (20%) patient. The first imaging test for all patients was a chest X-ray, and three (60%) patients underwent multiple chest X-rays before progressing to second-level imaging modalities. Three (60%) patients underwent an esophagram study, although only one (33.3%) was conclusive for an esophageal leak, whereas two (66.7%) were considered negative. All patients underwent computed tomography (CT), which provided a definitive diagnosis in all cases. Four (80%) CT scans were performed with oral contrast, all of which revealed extraluminal extravasation. Three (60%) patients had a pneumothorax, one of whom presented with tension pneumothorax. Figure 1 shows a CT scan performed for a perforation diagnosis. Two (40%) patients showed a radiological recurrence of hiatal hernia. Before consultation by our department, two (40%) patients had undergone chest tube placement immediately after receiving a diagnosis of esophageal perforation (one of them drained brown fluid and one drained purulent fluid), and one (20%) patient underwent endoscopic placement of a covered esophageal stent. The clinical presentations and imaging characteristics are summarized in Table 2.

### 3.4. Perforation Treatment

**Figure 2:**
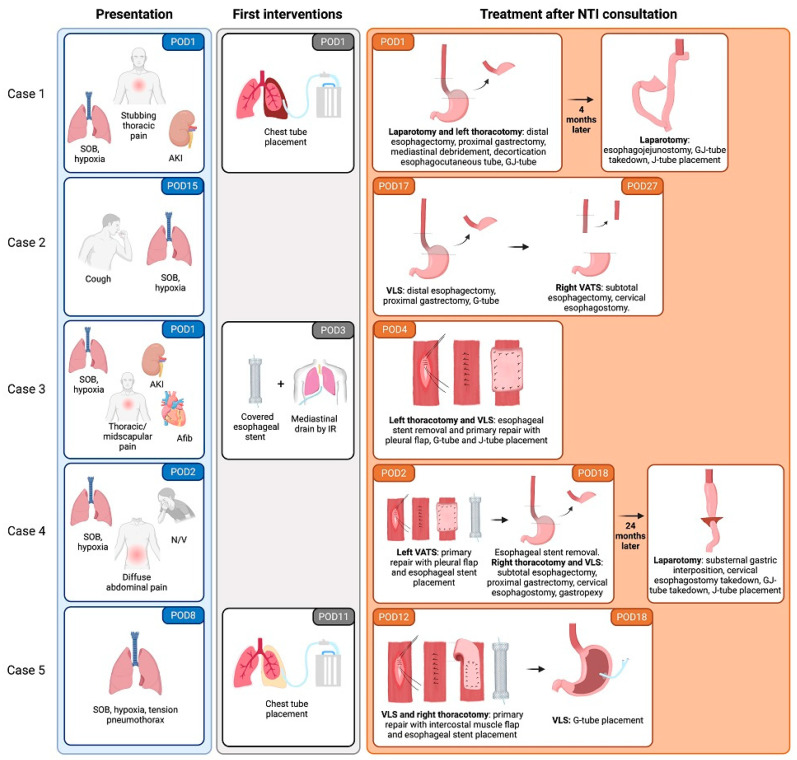
Summary of clinical presentation and therapeutic interventions in the study population. Note: Case 3 had an endoscopic GJ-tube placed between POD #2 and POD #18. Created with BioRender.com (accessed on 27 April 2025) (used with permission from Norton Thoracic Institute, St. Joseph’s Hospital, and Medical Center, Phoenix, Arizona). Afib: atrial fibrillation; AKI: acute kidney injury; G: gastrostomy; GJ: gastrojejunostomy; IR: interventional radiology; J: jejunostomy; NTI: Norton Thoracic Institute; N/V: nausea and vomiting; POD: postoperative day; SOB: shortness of breath; VATS: video-assisted thoracoscopic surgery; VLS: videolaparoscopy.

A summary of the clinical presentation and therapeutic interventions of each case is presented in Figure 2. The median time between index surgery and NTI consult was 4 days (IQR: 1.5–14.5; range 1–17), whereas the median time between clinical presentation and consultation by our department was 2 days (IQR: 0–3), ranging from 0 to 4 days. Four (80%) perforations were surgically or endoscopically identified in the distal esophagus, while one (20%) was located in the middle thoracic esophagus. Two (40%) patients (case 1 and 2) were treated with distal esophagectomy, proximal gastrectomy, and esophagostomy on postoperative day (POD) #1 and POD #17, respectively. The other three (60%) patients (cases 3, 4, and 5) underwent covered esophageal stent placement.

Case 3 had the esophageal stent placed on POD #3 before referral to our center. Our operative procedure included mediastinal drainage, stent removal, and primary repair with pleural flap via left thoracotomy along with laparoscopic gastrostomy and jejunostomy tube placement. The patient recovered without further surgical interventions.

Case 4 underwent left video-assisted thoracoscopic surgery (VATS), mediastinal abscess drainage, decortication, primary repair of the esophageal perforation with pleural flap, and esophageal stent placement on POD #2. However, 16 days later, the patient required re-exploration for a persistent infection and bilious output from the chest tube via a right thoracotomy. For this patient, a subtotal esophagectomy, esophagostomy, and proximal gastrectomy were performed.

Case 5 underwent right thoracotomy and explorative mediastinal abscess drainage, decortication, primary repair of the esophageal perforation with intercostal muscle flap, and endoscopic stent placement on POD #12, followed by a laparoscopic G-tube on POD #18.

In summary, all patients required both thoracic and abdominal exploration. One (20%) patient underwent a complete minimally invasive approach, three (60%) patients had a combination of laparoscopy and thoracotomy, and one (20%) patient underwent both open abdominal and thoracic surgeries. Overall, two (40%) patients recovered after primary repair, while three (60%) required limited esophagogastrectomy with esophagostomy. Immediate reconstruction was not attempted in any of the cases due to the patients’ poor clinical condition. Perforation treatment and postoperative course are summarized in Table 3.

### 3.5. Postoperative Course

The most common complication was altered mental status in three (60%) patients. Seizure and pneumonia were both recorded in another patient. One (20%) patient developed deep vein thrombosis, while another (20%) had thrombosis of the peripherally inserted central catheter line. One patient with acute kidney injury in the context of prior stage I chronic kidney disease required hemodialysis for 2 months and eventually stabilized with a creatinine level of 1 mg/dL. Three (60%) patients required tracheostomy placement due to persistent ventilator dependence. One (20%) patient experienced a persistent small leak, requiring a mediastinal drain, which was still in place at the 3-month follow-up. The median hospital stay was 58 days (IQR, 34–59), and the median surgical interventions performed during this time was 2 (IQR, 1–2). Upon discharge, all patients were transferred to a rehabilitation facility.

### 3.6. Outcome and Long-Term Follow-Up

The median follow-up was 14 months (IQR, 6–28), ranging from 3 to 98 months, with no deaths observed during this period. Of the three patients who underwent resection without reconstruction, two had already undergone reconstruction at follow-up: one (case 1) had an esophagojejunostomy 4 months later, while the other (case 3) underwent reconstruction with substernal gastric pull-up and takedown of the cervical esophagostomy 24 months after esophagectomy. All reconstructions were performed via laparotomy. The third patient with esophagectomy (case 4) was awaiting reconstruction at the 6-month follow-up. At the most recent visit, the median rating for nutritional status, digestive function, activity status, pain, and self-perceived well-being was 2 (IQR, 2–3), 2 (IQR, 2–2), 1 (IQR, 1–2), 4 (IQR, 4–4), and 3 (IQR, 2–3), respectively.

## 4. Discussion

Laparoscopic fundoplication is the most commonly performed antireflux surgical procedure for patients with GERD and/or hiatal hernia, and it is associated with excellent short- and long-term results; [5,6] however, early complications occur in about 5% to 10% of patients [15,16,17]. Gastroesophageal leaks, though rare, are among the most serious complications, affecting 0.1–0.3% of patients undergoing primary antireflux surgery in the modern era, a significant reduction from the 1% incidence observed in early experiences [10,18,19,20]. This study is one of the few case series focusing on missed iatrogenic injuries presenting as postoperative perforations after antireflux surgery, offering detailed information on presentation, diagnostic evaluation, and management. In addition, a literature review of comprehensively described cases of gastroesophageal perforation after LARS was conducted (Table 4). Although the review highlighted a significant knowledge gap regarding this severe complication, it allowed for the synthesis of some key information from the available reports.

Evidence suggests that the risk of postoperative leaks increases with the number of previous surgeries. For example, in a cohort of 940 patients stratified into four groups based on the number of prior antireflux procedures, Singhal et al. [19] observed a progressive rise in the incidence of postoperative leak rates, from 0.2% in patients undergoing primary ARS to 7.7% in those with three or more prior surgeries. While this cohort included patients undergoing more complex procedures such as Collis gastroplasty and Roux-en-Y reconstruction, which may elevate the risk beyond that of fundoplication alone, the trend underscores the cumulative surgical risk associated with redo procedures contributing to postoperative perforation.

Importantly, when such perforations are not recognized intraoperatively, they can lead to significantly worse outcomes. Postoperative leaks after antireflux surgery are rare, and have been associated with high morbidity and mortality, especially if the diagnosis is delayed [26,27]. In contrast, perforations detected and managed intraoperatively are more common, with some series reporting an incidence of over 10% in LARS, and are associated with lower morbidity [19,20,28]. Studies have shown that intraoperative perforations are far less likely to require reoperation than those detected postoperatively. For instance, Zhang et al. [29] reported a reoperation rate of only 2% for perforations treated during surgery, compared to a 75% for those discovered after the procedure. Additionally, postoperative perforations carry a remarkably higher mortality rate. In a series of 17 perforations following Nissen fundoplication, Schauer et al. [9] reported a 0% mortality rate for leaks identified and treated during surgery, compared to a 17% mortality rate for those diagnosed postoperatively. Similarly, Urschel et al. [10] reported a 17% mortality rate in their series of 12 perforations identified postoperatively. In our study, although the mortality rate was zero, leaks were associated with a substantial morbidity. More than half of the patients required multiple invasive procedures, contributing to extended hospital stays and prolonged recovery periods. This highlights the importance of intraoperative recognition and treatment of perforations, which may reduce the burden of this complication. Perforations detected intraoperatively are usually treated with primary repair, associated, in some cases, with different types of reinforcement (e.g., buttressing with wrap, intercostal muscle flap or pleura), or by endoscopic placement of clips or a covered stent. In most cases, no further treatment is required [28]. Timely diagnosis of perforations after surgery is crucial, as early recognition and intervention can significantly improve patient outcomes [9]. However, the non-specific clinical presentation of perforations often complicates diagnosis, as symptoms usually overlap with those of other normal postoperative conditions or complications, which translates into a low index of leak suspicion [30].

The severity and nature of perforation symptoms are influenced by the time elapsed between the iatrogenic injury and the diagnosis, as well as the site of the perforation [30,31]. In the context of antireflux surgery, esophageal perforations typically involve the distal thoracic or abdominal esophagus [9,10]. Distal esophageal perforations often present with retrosternal or interscapular pain, dyspnea, and cough. In contrast, perforations involving the abdominal portion of the esophagus or stomach are more likely to cause abdominal pain, along with nausea and vomiting. Moreover, perforations of the esophagus can also present with a combination of these symptoms, reflecting both abdominal and mediastinal contamination [30,32].

All of our patients were diagnosed with esophageal perforations with no gastric injuries. Perhaps because these patients, compared to those with gastric leaks, had a higher likelihood of being referred to a tertiary thoracic center, given the greater severity of their clinical condition. In fact, leaks involving chest contamination cause patients to present more critically. Urschel et al. [10] reported that patients with leaks confined to the peritoneum, as opposed to those with mediastinal contamination, tended to be less acutely ill. Similarly, all patients in our study presented with septic shock, highlighting the severity of mediastinal contamination. Fever was the most commonly reported symptom of perforation in the series described by Urschel et al. [10]. Interestingly, none of the patients in our series presented with fever.

In our cases, shortness of breath was the most common symptom, with hypoxia present in all patients. However, respiratory symptoms in surgical patients can be deceptive, given the overlap with expected postoperative status or pulmonary complications, such as pain induced tachypnea, atelectasis, pneumonia (as shown in this report), and pulmonary embolism. Urschel et al. [10] reported in their retrospective review that fever in two patients who later died from sepsis was initially attributed to respiratory causes, leading to a delayed diagnosis, which likely contributed to their poor outcomes. Moreover, patients presenting to the emergency department are typically evaluated for common medical issues, often overlooking potential surgical causes. Early consideration of iatrogenic causes, such as perforation, and prompt involvement of the surgical team in the work-up can help prevent delayed diagnoses and reduce the need for more invasive procedures.

Additionally, as shown in our series, the timing of symptom onset can vary, with some patients presenting within hours of surgery, whereas others may show signs of perforation several days after the procedure, further complicating early diagnosis. [10] Chest X-ray is usually the first imaging modality used, which can show indirect signs of gastroesophageal injury, including pleural effusion, pneumomediastinum, subcutaneous emphysema, pneumothorax, pneumoperitoneum, and atelectasis [33]. However, its low sensitivity and specificity limits its use in this context [34]. In most cases, an esophagram with Gastrografin is the next investigation performed. Some authors advocate for the routine use of a fluoroscopic study after antireflux surgery, which can detect recurrent hiatal hernias, evaluate whether the wrap is too tight, and assess gastric emptying [35]. However, its utility, especially in detecting visceral injuries, remains uncertain. In our cohort, an esophagram detected a leak in only one of three cases investigated. This is consistent with a study by Tsunoda et al. [36], who reported a positive esophagram in only 20% of patients with a postoperative leak. Contini et al. [37] observed that routine esophagram 24 h after antireflux surgery failed to recognize the only perforation (located at the fundus) in 92 patients. Robertson-More et al. [38], investigating 391 patients who underwent paraoesophageal hernia repair, observed that routine upper GI contrast studies within 48 h of surgery changed the management in 0.8% of cases and were unhelpful in determining the need for early reoperation. Given the lower sensitivity and specificity of swallow studies compared to CT, along with the need for patient cooperation, their use in emergency situations is limited and should not be encouraged [33,39]. CT with oral and intravenous contrast, providing both a sensitivity and specificity > 90% for gastrointestinal perforations, can be particularly helpful in distinguishing rare conditions, such as postoperative leaks, from more common clinical situations, like pneumonia or pulmonary embolism [33,40]. CT can be valuable in case of rapidly deteriorating clinical conditions, providing a timely and accurate diagnosis. It commonly reveals pleural effusion, pneumomediastinum, sometimes with air–fluid level, pneumothorax, subcutaneous emphysema, and contrast extravasation [41,42].

Early diagnosis significantly increases the likelihood of successful conservative or minimally invasive surgical treatment. In our series, the median time from index surgery to presentation to our service was 4 days (range, 1–17), which likely contributed to the extent of mediastinal contamination and tissue necrosis observed. As a result, extensive surgical procedures, such as esophagectomy and gastrectomy, were required in most of these cases, leading to high morbidity.

Conservative management is always preferred over radical surgical interventions for esophageal perforations. For example, minimally invasive endoscopic treatments, such as stenting, suturing, or vacuum-assisted closure, often combined with radiologically guided drainage, are proven successful strategies for iatrogenic perforations and post-anastomotic leaks, which are usually diagnosed early. In their review of 32 endoscopic iatrogenic perforations, Montminy et al. [43] reported a median time from injury to diagnosis of 2 h. Contained and small perforations, especially those in the peritoneal cavity, diagnosed in patients who are not acutely ill, are most suitable for conservative management, which consists of broad-spectrum antibiotics, nasogastric tube placement, enteral or parenteral nutrition, and the cessation of oral intake along with percutaneous drainage if the source control of infection is not adequate [10,33].

Esophageal perforations that are diagnosed early can also be treated with endoscopic stent placement, although those located near the esophagogastric junction are more likely to fail [32,33,44]. In cases of mediastinal contamination, esophageal stenting may be combined with percutaneous or thoracoscopic drainage to achieve better infection control. Additionally, endoscopic vacuum therapy, initially described for anatomic leaks, has recently been investigated for acute esophageal perforations with promising results, particularly when the diagnosis is made within 24 h [45,46]. These conservative and minimally invasive techniques are suitable only for clinically stable patients.

Surgical intervention is most commonly required when the diagnosis is delayed beyond 24 h due to the increased probability of extensive mediastinal or peritoneal contamination and the resulting critical condition of the patient [32]. When the diagnostic and therapeutic delay exceeds 24 h, the mortality rate is at least two times higher, and prompt intervention is essential to improve outcomes [47]. Primary repair, with or without reinforcement, may be attempted if necrosis is limited; otherwise, resection is advisable to avoid further complications, with delayed reconstruction considered for unstable patients, allowing time for stabilization before attempting more complex surgical repairs. The high morbidity associated with postoperative perforations underscores the importance of intraoperative recognition and treatment, or at least early diagnosis as well as timely intervention, to ensure the best possible outcome.

Given the high morbidity associated with undetected leak after LARS, we believe that a collaborative effort should be made to reduce the incidence of intraoperative perforations and virtually set to zero the occurrence of missed gastroesophageal injuries. Along the same lines of the SAGES safe cholecystectomy program [48], a comprehensive list of recommendations could be developed to prevent, or at least help recognize and intraoperatively manage, gastroesophageal perforations during antireflux surgery. Such guidelines could serve as an invaluable resource to guide surgeons toward a decisive enhancement in LARS safety.

This study has some limitations. First, cases were retrieved from the personal records of all surgeons at our department. While we made efforts to comprehensively include all relevant patients, it is possible that some cases were inadvertently missed, introducing potential selection bias. Second, the retrospective nature of this study may limit control over confounding variables and restrict the ability to establish causality, while the small sample size (n = 5) reduces statistical power and external validity, thereby potentially affecting the generalizability of the findings. Nonetheless, given the condition under investigation (i.e., a very rare complication after antireflux surgery), a case series remains the most practical and informative study design. Unlike the case report format, aggregating five comparable cases allows for the recognition of patterns in presentation, surgical management, and outcomes. This synthesis offers clinically relevant insights that cannot be derived from isolated case descriptions and contributes to a more comprehensive understanding of this uncommon but serious complication. Moreover, the medical literature does not establish a universally accepted minimum number of cases required for a case series [49,50]. For these reasons, we believe that a case series design is more appropriate than a case report format and justifies the classification of this manuscript as an original article.

## 5. Conclusions

Our case series emphasizes the importance of a high index of suspicion for perforation in post-LARS patients as clinical presentation, like hypoxia and sepsis, can overlap with diverse clinical manifestations and other postoperative complications. Additionally, the delayed diagnosis in some of our cases underscores the need for early testing for leaks and timely surgical consultation. Prompt recognition may allow for conservative management or less invasive interventions, and endoscopic treatment, with or without drainage, or primary repair are viable options in the absence of advanced contamination or necrosis. In light of the potentially catastrophic consequences of unrecognized leaks, creating clear guidelines on injury prevention and early recognition could significantly enhance LARS safety.

## Figures and Tables

**Table 1:** Study population characteristics.

Demographic and Clinical Characteristics	Study Population (N = 5)
Age, years	73 (67–74)
Gender	
Female	4 (80)
Male	1 (20)
BMI, kg/m^2^	31.60 (30.30–36.11)
**Antireflux surgery**	
Surgery indication	
PEH	3 (60)
GERD and PEH	2 (40)
Redo surgery	0
Approach	
Laparoscopic	3 (60)
Robot-assisted	2 (40)
Fundoplication type	
Nissen	3 (60)
Toupet	1 (20)
Watson	1 (20)
Mesh used	4 (80)
OR time, minutes	210 (170–235)

Data are expressed as no. (%) or median (IQR). BMI: body mass index; GERD: gastroesophageal reflux disease; OR: operative room; PEH: paraoesophageal hernia.

**Table 2:** Clinical presentation and imaging characteristics in the study population.

Clinical Presentation	Study Population (N = 5)
Time from LARS to clinical presentation, days	2 (1–8)
Shortness of breath	5 (100)
Pain	3 (60)
Abdominal	1 (33.3)
Thoracic	1 (33.3)
Thoracic–midscapular	1 (33.3)
Hypoxia	5 (100)
Septic shock	4 (80)
Emergent intubation	4 (80)
Atrial fibrillation	1 (20)
Acute kidney injury	3 (60)
Subcutaneous emphysema *	3 (60)
WBC count, × 10^9^/L	14.5 (13.8–16.5)
**Imaging**	
Chest X-ray	4 (80)
Pleural effusion	4 (100)
Pneumothorax	3 (75)
Pulmonary consolidation	2 (50)
Esophagram	3 (60)
Perforation	1 (33.3)
No perforation	2 (66.7)
Computed tomography	5 (100)
Contrast extravasation **	4 (80)
Pleural effusion	5 (100)
Pneumomediastinum	5 (100)
Pneumothorax	3 (80)
Hiatal hernia recurrence	2 (40)

Data are expressed as no. (%) or median (IQR). LARS: laparoscopic antireflux surgery; WBC: white blood cell. * Clinical and/or radiological diagnosis. ** Four patients had CT scan with oral contrast, and all of them showed extravasation.

**Table 3:** Perforation treatment and postoperative course.

Treatment and Postoperative Course	Study Population (N = 5)
Time from clinical presentation to NTI consult	2 (0–3)
Perforation location	
Distal esophagus	4 (80)
Middle thoracic esophagus	1 (20)
Primary repair *	2 (40)
With intercostal muscle buttressing and esophageal stent	1 (50)
With pleural flap	1 (50)
Esophagectomy	3 (60)
Proximal gastrectomy	3 (60)
Gastrostomy or gastrojejunostomy tube	5 (100)
Jejunostomy tube	1 (20)
Esophagostomy	3 (60)
Complications	5 (100)
Pneumonia	1 (20)
Seizure	1 (20)
Altered mental status	3 (60)
Deep vein thrombosis	1 (20)
PICC line thrombosis	1 (20)
Ventilator dependence requiring tracheostomy	3 (60)
No. of surgeries **	2 (1–2)
Hospital stay, days	58 (34–59)
Follow-up, months	14 (6–28)

Data are expressed as no. (%) or median (IQR). NTI: Norton Thoracic Institute; PICC: peripherally inserted central catheter. * Excluding cases of primary repair necessitating further surgical treatment within 30 days. ** Surgical interventions required before discharge.

**Table 4:** Summary of studies reporting perforations after laparoscopic antireflux surgery.

Author, Year	N	Indication	Procedure Type	Symptoms	Perforation Location	Treatment	Postoperative Course	LOS (Days)	Outcome
Urschel et al., 1994 [10]	11	GERD (3 redo)	Modified Nissen (n = 9); Belsey (n = 1); intrathoracic Nissen (n = 1)	Fever (n = 10)	Chest (n = 6); abdomen (n = 5)	Abdominal: nonoperative (n = 4); esophageal repair (n = 1). Chest: nonoperative (n = 1); thoracotomy drainage (n = 2); transthoracic esophageal repair (n = 2); gastrectomy (n = 1).	NR	NR	2 deaths (sepsis), 1 after thoracotomy drainage and 1 after transthoracic esophageal repair. Follow-up time not reported.
Schauer et al., 1996 [9]	6	GERD	Laparoscopic Nissen	Abdominal pain (n = 4); chest pain (n = 1); respiratory distress (n = 1)	NR	Wrap takedown, primary perforation closure and rewrap (n = 5); one of them had also omental patch and J-tube. Wrap takedown, primary closure, excision of the necrotic stomach, G-tube, duodenal feeding tube, and abdominal drainage (n = 1).	NR	14 (range, 10–32)	17% mortality at 11-months of follow-up
Pohl et al., 2001 [21]	1	GERD	NR	NR	Esophagus	Surgical drainage and redo Nissen	NR	NR	NR
Lindenmann et al., 2015 [22]	1	NR	NR	NR	3 mm esophageal leak proximal to the fundoplication cuff	Endoscopic fixation using OTSC and self-expandable, fully covered removable stent	Stent dislocation, repositioning using purse string; sepsis on day 5; hematemesis from esophageal-aortic fistula at clip site on day 6	NR	NR
Wang et al., 2016 [23]	1	Hiatal hernia	Toupet	Fever on POD4, acute chest pain and respiratory distress on POD7	2.5 cm distal esophageal perforation	Primary repaired with 4-0 Prolene continuous sutures	10 days after repair, suture disruption at EGD; paraoesophageal tube pulled into the esophagus endoscopically + NGT positioning	57	Oral nutrition was tolerated without sequelae; well at 6-month follow-up
Pamart et al., 2021 [24]	1	GERD (redo)	Nissen	POD4: abdominal pain with rebound tenderness, nausea, dysphagia, fever, sepsis	Gastric perforation on the wrap	Nissen dismantling and primary suture	Subphrenic abscess resolved with antibiotics	22	NR
Anthony et al., 2024 [25]	1	NR	Nissen, Posterior cardiopexy + anterior gastropexy	POD7: tachypnea, tachycardia, rebound tenderness	EGJ	Exploratory laparotomy, endoscopic clips; AbThera dressing; definitive closure 48 h later	Fever, ileus	13	Resumed regular diet, no further complications. Follow-up time not reported.

Abbreviations: EGD: esophagogastroduodenoscopy; EGJ: esophagogastric junction; GERD: gastroesophageal reflux disease; LOS: length of (hospital) stay (days); N: number of patients; NR: not reported; OTSC: over-the-scope clips; POD: postoperative day.

## Data Availability

The data presented in this study are available upon reasonable request to the corresponding author. The data are not publicly available due to institutional confidentiality agreements.

## Supplementary Materials

The following supporting information can be downloaded at: https://www.mdpi.com/article/10.3390/jcm14134577/s1, Figure S1. Literature review flowchart.
