# Supplementary figures and images for: Missed Gastroesophageal Injuries During Antireflux Surgery: Infrequent but Catastrophic Complications
Source: J Clin Med. 2025 Jun 27;14(13):4577. doi: 10.3390/jcm14134577 (PMC12249958; doi:10.3390/jcm14134577)

**Figure S1.** Literature review flowchart.

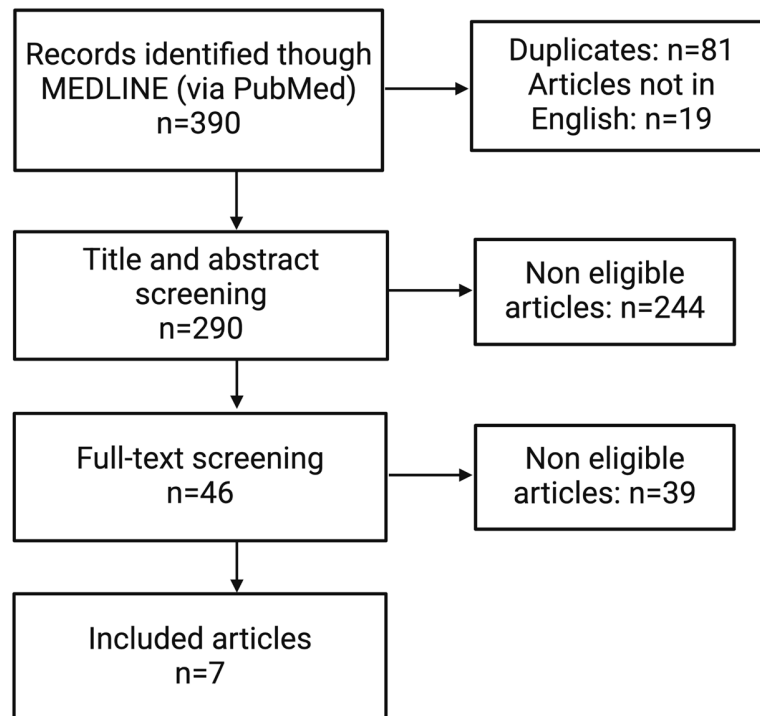

Supplement: Supplementary file 1 [file jcm-14-04577-s001.zip › jcm-3663403-supplementary.pdf]
